# Elements of Materia Medica and Therapeutics

**Published:** 1833-04-01

**Authors:** 


### Elements of Materia Medica and Therapeutics. By *A. T. Thomson*, M.D. Vol. I. pp. 747. October, 1832.

The Materia Medica, after all, furnishes us with the powder, shot, and cannon by which we assail the citadels of disease, and, without this *materiel* of war, all our fine-spun rules of gunnery and fortification are of no avail. Dr. Thomson has dedicated a long professional life, not, indeed, to the exclusive study of this branch of our art, but certainly to a more than ordinary cultivation of it:—he is, therefore, well qualified to bring this portion of study to the greatest degree of perfection of which it is, at present, susceptible. He laments that a wrong direction is given, by the corporate bodies, to the routine of the tyro's studies, by compelling him to *commence* his medical education with the materia medica; whereas he should be directed previously to attend at least one course of natural history, botany, chemistry, anatomy, and physiology, without some knowledge of which, he cannot comprehend the doctrines delivered in a course of materia medica, far less those relating to therapeutics. The author flatters himself that, in this work, he has rendered the mistake in medical education sufficiently evident, and, consequently, tended to effect its correction. Dr. Thomson appears to have availed himself, with his characteristic assiduity, of the labours of the Continental chemists and medical writers, as well as of his own country and America, to enrich his work; and, although much of it must necessarily be a compilation, yet the author has introduced the results of 30 years' attentive observation in the treatment of disease into the work, and thereby given a considerable portion of original information to his readers.

This first volume is divided into three parts—first, on the general action of medicines—secondly, on the classification of medicinal agents—and, thirdly, on materia medica and therapeutics. It must be evident that such a work is insusceptible of analysis, and not a fair subject of criticism. The latter, indeed, is out of the question, for he must be cynical, who would dream of assailing so careful and valuable a publication. Whichever way we turn, we are forcibly struck with the professional enthusiasm, professional information, and general knowledge of the estimable author. Such teachers confer as much credit on an university, as it can possibly reflect on them.

We should be loth to indulge in mere commendation, without placing before our readers a few specimens of the manner in which the subjects are handled. We will endeavour to comprise, in this necessarily brief notice, some passages illustrative of the style, the taste, the learning, and the arrangement of Dr. Thomson. We have said that the first part is devoted to the consideration of the General Action of Medicines. This is divided into three sections. The first is devoted to their chemical, revulsive, direct action, and so on—the second, to their action on the solids, the fluids, and the functions—the third to the investigation of the circumstances modifying their action. The first quotation in which we will indulge, shall contain some of our author's observations on the action of medicines decomposed in the stomach, or after entering the system. This affords a sample of his pharmaceutic physiology.

"With respect to vegetable medicines, this decomposition is effected in the same manner as that of alimentary substances. Many experiments have ascertained the fact, that all simple aliments, such as *albumen*, *gelatin*, *fibrine*, *caseous* matter, *vegetable mucus*, *sugar*, *starch*, and *gluten*, are soluble in the gastric juice; and that all compound aliments, containing these substances as constituents, are also dissolved by it, under certain conditions, connected with the structure of the compound substances. A substance, for instance, the components of which are, when separate, readily soluble in hot water, is very quickly dissolved in the gastric juice. It is less quickly dissolved if the components, separately, require the assistance of acids for their solution; as those, for instance, which contain much *gluten*, *concrete albumen*, *fibrine*, and *caseous matter*: and the gastric juice scarcely acts at all upon the *husks of grains*, the very *hard fibres of woody plants*, *hairs*, *feathers*, and substances of a very compact texture. Now, as all vegetable substances are components of some of these simple aliments, and are more or less susceptible of solution in the stomach, it is evident that all vegetable medicines must also undergo digestion to a certain extent; although it is probable that some of them previously act upon the nerves of the stomach. The decomposition, however, is generally first effected, and the active principle being thus extricated, acts either *directly* upon the nerves of the stomach, or escapes into the circulation, whilst the other constituents are completely digested. It is to this circumstance that we may ascribe the time which elapses, after swallowing some medicines, and the period when their operation becomes apparent. Thus, if half a drachm of the powder of the root of Ipecacuanha be swallowed, from fifteen to thirty minutes generally elapse before vomiting is produced, a circumstance which we may fairly attribute to the envelopment of the *Emetina*, the active constituent of the Ipecacuanha, in the *Wax*, *Gum*, *Starch*, and *ligneous* matter of the root: it cannot exert its influence until extricated from these by the process of digestion. In this manner, also, *Veratria*, the active ingredient of Colchicum; *Strychnia*, that of Nux-vomica; and the medicinal principles of many other substances; are separated and pass into the circulation. The decomposition of medicines in the stomach by the digestive function, is not, however, confined to vegetable matters; the Acetas Potassæ, and some other salts in which vegetable acids enter as components, are liable to undergo the same process; the acid is digested, whilst the alkali passes into the circulation, and is excreted by the kidneys. Such is the result of the digestive process on many medicines: those parts which are readily soluble in gastric juice are digested; whilst the active principle is set free, remains unaltered, and exerts its influence on the system. In all these, and similar instances, the decomposition is effected by the vital powers of the stomach; and the active principle of the medicine which is eliminated either acts *directly* upon the stomach, extending its influence to distant parts of the system, or it is absorbed and carried, in the course of the circulation, to those organs on which its appropriate action becomes apparent, whether *diuretic, sudorific, expectorant,* or otherwise." 20.

The effect of climate on the properties of drugs occupies our author's attention. From the midst of many curious and interesting remarks we copy the following.

"The effect of climate upon the medicinal properties of plants is strikingly illustrated in the history of the Meadow Saffron, Colchicum *Autumnale.* In England and many other countries, the Colchicum always contains an acrid, alkaline, bitter principle, *Veratria,* of great importance as a remedy, and a virulent poison when overdosed. At some seasons of the year, the bulb of the Colchicum, in this country, is more active than at others: but during the whole year, it contains a sufficient portion of its medicinal principle to render it a very hurtful substance if eaten as food:—yet, in other parts of the globe it may be eaten with impunity at some seasons. Kraterhvill, a German author, in his work 'de Colchico,' relates instances in which entire bulbs were eaten without any bad effect being produced on the habit. Krapf, another German writer, says that he has eaten the bulb with impunity in autumn, and that it is then eaten in Carniola and Istria: yet autumn is the period of the year when the bulb is most active in this country. The celebrated Haller, also, avers that it is both tasteless and inert in autumn. Now to what are we to ascribe those peculiarities in the Colchicum of the countries in which these writers lived? Their veracity is undoubted; yet their relations are at direct variance with our experience in this country, and, therefore, we can only ascribe the difference to climate and to local circumstances. For the same reason, Senna transported from Upper Egypt and grown in the South of France varies both in the external characters of the leaf and in its purgative properties: the leaves are more obtuse, less bitter and less nauseous when chewed, and much less purgative than the Egyptian Senna. The tree named Myrospermum *Frutescens,* when it grows in New Grenada, yields *Balsam of Tulou;* but when it grows in Peru it yields a very different Balsamic substance, which, from the place of its production, has been named *Balsam of Peru.* I may also mention that Mint, and many other plants which yield an essential oil, afford it of a much less penetrating odour in the South of Europe than in England: and, it is a curious fact, that almost all strong smelling plants lose their odours in a sandy soil." 72.

As an instance of the general knowledge brought to bear upon the matter of the work by Dr. Thomson, we present our readers with his view of the influence of superstition on medicines, or rather on their operation.

"The influence of *Superstition* over the operation of medicines is much more limited than that of Credulity; but it operates as a powerful obstacle to the advancement of medicine in those countries where it still exists. At an early period in the history of society, Superstition supplied many articles of the Materia Medica; but, as education advanced, these fell into disuse. The influence of Superstition arose from the characters of Priest and Physician being combined in the same person. This was the case with the Jews, as we learn from the Mosaical accounts of their early history. The priests of Æsculapius were also the first physicians of the Greeks. The Druids were those of the Northern nations: and, in the history of our own country, we read that, after the Anglo-Saxons had embraced the Christian religion, the clergy were the only medical practitioners. The first medical book translated into the Saxon language was the work of Apuleius on the virtues of herbs; and on this the whole of the practice of medicine was founded until the tenth century, when the monks took up both the teaching and the practice of the healing art, and drew their information from the writings of Galen, Rhazes, Avicenna, and other Arabians, which were translated into Latin and deposited in the monasteries. In the eleventh century, the clergy applied themselves particularly to the study of Materia Medica. Richard Fitz-Nigel, who died Bishop of London, A.D. 1198, had been apothecary to Henry the Second: Roger Bacon, who flourished in the thirteenth century, practised physic, although a monk: in the same century, Nicolas de Farneham, physician to Henry the Third, was made Bishop of Durham: and many other doctors of medicine were, at various times, elevated to ecclesiastical dignities. It was even thought essential that physicians should remain in a state of celibacy; and it was not until the fifteenth century that they were permitted to marry in countries professing the Roman Catholic religion. In periods like these Superstition held her sway, equally over the religious opinions and the credulity of the multitude, in matters relating to the cure of their diseases. It was easier for a crafty priesthood to work upon the weakness of the mind than to investigate the nature of diseases and their remedies; thence, we find that the same means which they had so successfully employed in duping mankind, in matters of religion, were turned to equal account in what related to medicine. Little confidence was placed in medicines, even at a later period; for Burton, in his Anatomy of Melancholy, informs us 'that there was of old no use of physicke amongst us, and but little at this day, except it be for a few nice idle citizens, surfetting courtiers, and staufed gentlemen lubbers. The country people use kitchen physicke.'

If we go back to the period of Scripture history, we find that the Jews had great faith in phylacteries—a species of amulets, which are still held in esteem in India and other Eastern countries. They consisted of portions of Scripture written upon vellum of a shape and size adapted to the part of the body on which they were to be worn. Thus, a phylactery for the head, preserved in the Duke of Sussex's library, consists of four slips of vellum, with verses from Scripture written on each. These are separately rolled up, and placed in a small leathern bag, upon which is written the word *schin*. It is tied to the head by means of thongs of leather, so as to permit the bag to rest on the forehead. These phylacteries are called tephillin, shel-rash, tiffila of the head. Those of the arm are called sheljad: they are written upon a strip of vellum, which is rolled up spirally to a point, and enclosed in a case made of the skin of any clean beast. They are bound upon the arm, in a situation as near to the heart as possible, by a thong which must go seven times round the arm in a spiral manner and terminate by being wound three times round the middle finger. Coral worn round the neck was supposed to possess the power of driving away evil spirits.*

As Christianity advanced, the Clerical physicians seemed anxious to render the dogmas of the existing religion subservient to medicine; and, consequently, relics were introduced into the Materia Medica. We read, that bread dipped in oil at the shrine of St. Anthony, at Rome, was believed to prevent Hydrophobia in those bitten by a rabid animal; that a ring, taken from the body of St. Remigius, and dipped in water, produced a drink very efficacious in fevers; and the

---

"It is melancholy to think that a relic of this superstition is still countenanced by the higher ranks in this country: beeds formed of the root of the Bryony are strung together, and sold under the name of anodyne necklaces, for facilitating the protrusion of the teeth in the gums of an infant."

following cure for Epilepsy in children is recommended by John of Gaddesden, Walter Gilbert, and others, who flourished in the thirteenth century. 'When the patient and his parents have fasted three days, let them conduct him to the church. If he be of a proper age, and in his right senses, let him confess. Then let him hear mass on Friday, during the fast of *quatuor temporum*, and also on Saturday. On Sunday, let a good and religious priest read, over the head of the patient in the church, the gospel which is read in September, in the time of vintage, after the feast of the holy cross. After this, let the priest write the same gospel devoutly, and let the patient wear it about his neck, and he shall be cured. The gospel is—' This kind goeth not out but by prayer and fasting.' " 86.

The Second Part comprises the Natural Classification of Medicinal Agents. The first section is devoted to the consideration of Animal, the second to that of Vegetable Substances. A short extract will shew Dr. Thomson's mode of treating this division of his subject.

"Vegetable Substances.

Although the Linnean or Artificial System be that which has hitherto been employed for classing medicinal plants, as objects of Botany, yet, the Natural System holds out so many advantages to Medical Science, that there can be one opinion only of its superiority in a practical point of view: it informs the medical enquirer not only of the botanical affinities of the plants, but also supplies him with a knowledge of their properties and qualities. This acquaintance with the properties of even one plant of any order enables him to form some idea of the remedial value of all the other plants in the same order, and, if needful, to substitute upon fixed principles, any one of them for that which is more usually employed.

The great divisions of this System are *two:*

I. Vascular plants, Vasculares; plants having spiral vessels, both in the stems and leaves; cuticular stomatia;* distinct flowers; and sexual organs.†

II. Cellular plants, Cellulares; plants devoid of spiral vessels and sexual organs.

The first division contains, with a few exceptions, all the plants comprehended in the Materia Medica of the British Colleges. It is subdivided into two subclasses:

1. Dicotyledonous plants, or Exogenæ; plants with distinct pith, wood, and bark, in the stem and its ramifications; reticulated leaves; and fruit having an embryo with two or more opposite cotyledons or seed lobes. The plants grow by new layers annually deposited on the exterior surface.

2. Monocotyledonous plants, or Endogenæ; plants with no distinction of pith, wood, and bark, in the stem; with leaves displaying parallel veins; and fruit having an embryo with one cotyledon only; or, if there be two cotyledons, they are not opposite but alternate. The increase of the stem is by internal additions.

Seven eighths of the medicinal plants in the British Pharmacopœias belong to the first of these subclasses; as will appear in the following arrangement of them under the orders.

DYCOTYLEDONS.

Umbelliferæ.—Herbaceous plants, with fistular-furrowed stems, and divided

---

* "Stomatia—organized pores, perhaps for breathing, like the spiracula of insects."

† "Sexual organs comprehend the anthers and stigma."

or simple leaves sheathing at the base : *flowers* in umbels :* the *calyx* entire or five-toothed ; *petals* 5, alternate with 5 *stamens* incurved in æstivation : *ovarium* didymous,† with two *styles* and solitary pendulous *ovula.* The *seed-vessel* is traversed with vertical ridges ; and has, in some instances, near the base, *vittæ,* or linear receptacles of oil.

*Geographical position :* The northern parts of the northern hemisphere.

*Yielding an aromatic volatile oil* :

Carum *Carvi.*
Pimpinella *Anisum.*
Fœniculum *vulgare.*
Daucus *Carota.*
Coriandrum *sativum.*
Anethum *graveolens.*

*an aromatic principle :*

Archangelica *officinalis.*

*Ammoniacum :*

Ferula *Orientalis.*

*a fetid gum-resin :*

Opopanax *Chironium.*
Ferula *Persica.*
Ferula *Assafœtida.*
Bubon *Galbanum.*

*an acrid gum-resin :*

Cuminum *Cyminum.*

*a narcotic principle* :

Conium *maculatum.*" 99.

Part III, treats professedly of the Materia Medica and Therapeutics. We have in order, Vital Agents—Excitants—Sedatives—Refrigerants—Narcotics—Antispasmodics—Tonics. And here we must notice our Author's Table of Classification. Almost every writer on the Materia Medica has had his own, and rejected those of his predecessors. The fact is, that it is impossible to frame a classification quite free from objections, for many drugs act on several organs, and not on one individually. We might point out many parts of Dr. Thomson's Classification which are open, critically speaking, to serious objections, but believing, as we do, that a faultless classification is altogether utopian, we think it scarcely worth while to dilate upon almost unavoidable defects. In fact we are no great sticklers for classifications, and the sense of the age, if we mistake not, is opposed to the encouragement of these cobwebs of science. Men have now discovered that the true study of nature is the study of facts, and that all generalizations should be strictly inductive. A general rule is in its proper sense an algebraic expression, comprising a certain number of particular cases. Classifications are useful only as generalizations, as comprising an almost indefinite number of facts within definite and determinate limits. The most simple classification is therefore the best, for it is only of use as leading the mind to the more easy acquisition of the facts embraced in it. Provided then that a classification be devoid of intricacy and confusion, that it arranges the subjects at issue in a natural order, and that where it generalizes

* "Umbel—that form of inflorescence in which the subordinate stalks, rising on the summit of a common flower stalk, extend in rays."

† "Two joined together."

it does so fairly and not hypothetically, we ourselves are perfectly satisfied. The complicated systems of nosology, and those artificial arrangements that attempt to usurp the very throne of nature, and delude the mind by their shadowy forms, are fast sinking before the increased intelligence of the age. The following, without further preface, is the classification of Dr. Thomson.

" TABLE OF CLASSIFICATION.

I.—VITAL AGENTS.

A.—Influencing the body generally;

*a.*—by operating directly on the nervous system :

* *increasing action.* — Excitants.

* * *diminishing action* { *Primarily* { Sedatives. Refrigerants. } *Secondarily* { Narcotics. Antispasmodics. } }

*b.*—on the muscular and sanguiferous systems :

Tonics.
Astringents.

*c.*—on the secerning system :

Errhines.
Sialogogues.
Expectorants.
Emetics.
Cathartics.
Diuretics.
Emmenagogues.
Diaphoretics.

B.—Influencing the body solely by their action on the part to which they are applied.

Epistastics.
*a. Rubefacients.*
*b. Vesicants.*
*c. Actual Cauterants.*

II.—CHEMICAL AGENTS;

A.—Influencing the state of the body, or its contents, by their chemical properties :

* *acting on the surface.* — Escharotics.
*a. Potential Cauterants.*

* * *on the contents of cavities.* { Antacids. Antalkalies. *a. Antiseptics.* Antilithics. }

III.—MECHANICAL AGENTS :

Demulcents.
Diluents." 164.

We never pretended to offer a review of Dr. Thomson's work. Comprising much new matter with more that is merely elementary, it is too much to expect that a reviewer can peruse such a work sufficiently to point out all that is new, or all that is deserving of particular attention. Yet he may satisfy himself of its general tenor, and may be able to determine its general merits or defects. In this manner we have judged the present volume, and we think we may safely pronounce it creditable to the author, creditable to the school of which he is no inconsiderable ornament, and creditable to the character of British medicine. Need we say more, need we add the hackneyed recommendation to our students to buy? Most assuredly a good elementary work is certain of being successful in the end, and such a work we consider the present.

